# Standardizing oral microbiome sampling for qPCR: methodological and exploratory insights into nutritional status

**DOI:** 10.1038/s41598-026-43909-7

**Published:** 2026-03-14

**Authors:** Karina Mendes, Ana T. P. C. Gomes, Cristina Maria Mendes Resende, Isabela S. Ribeiro, Rafael M. C. Oliveira, Nuno Rosa, Maria T. C. Muniz, Maria J. Correia

**Affiliations:** 1 Center for Interdisciplinary Research in Health (CIIS), Faculty of Dental Medicine (FMD), Universidade Católica Portuguesa, Estrada da Circunvalação, 3504-505 Viseu, Portugal; 2 Graduate Program in Hebiatria, University of Pernambuco, Recife, Brazil; 3 Graduate Program in Molecular and Cell Biology, University of Pernambuco, Recife, Brazil; 4 Laboratory of Molecular Biology, Hospital Universitário Oswaldo Cruz, University of Pernambuco, Recife, Brazil

**Keywords:** Oral microbiome, Sampling methodology, Saliva, QPCR, *Bacillota*, *Bacteroidota*, Biological techniques, Biomarkers, Diseases, Health care, Medical research, Microbiology

## Abstract

Standardization of oral sample collection methods is essential for accurate and reproducible microbiota quantification. This methodological study aimed to evaluate different oral collection methods to identify the most consistent approach for bacterial quantification by qPCR using samples from adolescent individuals. In addition, to assess the biological applicability of the best method, an exploratory analysis compared bacterial profiles between eutrophic and overweight/obese adolescents and explored associations between bacterial abundance and body composition parameters. Samples of unstimulated saliva, cheek swabs, and biofilm were collected from the same individuals, and qPCR was used to quantify total bacteria (16 S rRNA gene), *Bacillota*, and *Bacteroidota* phyla. Unstimulated saliva produced the lowest variability in bacterial quantification compared with other methods (*p* < 0.05). Moderate correlations were observed between saliva and biofilm, whereas saliva and cheek swab showed weak associations. Although bacterial copy numbers tended to be higher in overweight/obese individuals, these differences were not statistically significant. Correlation matrices suggested group-specific associations between bacterial taxa and body composition parameters, demonstrating the potential of saliva for microbiome assessment in studies of nutritional and metabolic health. This study validated unstimulated saliva as a reproducible, non-invasive, and cost-effective biofluid for oral microbiota quantification by qPCR. The method provides consistent results suitable for large-scale, translational, or point-of-care applications.

**Supplementary Information:**

The online version contains supplementary material available at 10.1038/s41598-026-43909-7.

## Introduction

The human microbiome, particularly the communities residing in the oral cavity and gastrointestinal tract, plays a fundamental role in maintaining host health. These microbial ecosystems contribute to essential physiological processes such as nutrient metabolism, immune modulation, and epithelial barrier maintenance^1^. Dysbiosis—an imbalance in microbial composition—has been increasingly implicated in a range of chronic conditions, including obesity^2^, type 2 diabetes^3^ and systemic inflammatory diseases^4^. As a result, microbiome profiling has emerged as a powerful approach for understanding host-microbe interactions and for identifying biomarkers associated with health and disease^5^. Currently, there is growing interest in harnessing microbiome profiles for diagnostic purposes, clinical monitoring, and translational research^6^. To be clinically useful, however, microbial measurements must be accurate, sensitive, and reproducible^7^. Reliable microbiome analysis depends on minimizing technical artifacts and biases introduced by sampling methodologies. Among the available molecular techniques, quantitative polymerase chain reaction (qPCR) has become a popular tool for microbiome profiling^8,9^. qPCR is cost-effective, highly sensitive, and allows for the targeted quantification of both total bacterial load—often through amplification of the 16 S rRNA gene—and specific bacterial taxa of interest. Despite its strengths, qPCR outcomes are critically dependent on upstream factors such as sample collection, DNA extraction, and overall sample integrity^10^.

In oral microbiome studies, the method of sample collection is a critical determinant of data quality and biological relevance^11^. Unlike the gut microbiome, where stool is a relatively standardized sample type, the oral cavity presents multiple sampling options—such as saliva, mouthwashes, buccal or tongue swabs, and dental biofilm (plaque). Each of these sample types captures distinct microbial niches and can vary widely in terms of microbial load, diversity, and taxonomic composition^11^.

Saliva is frequently used due to its ease of collection and its representation of a pooled microbial signal from various intraoral sites. However, it may dilute signals from site-specific communities^12^. Swabs, often applied to the tongue dorsum, buccal mucosa, or gingival margin, provide more localized microbial profiles but are susceptible to variability in technique and sampling pressure^13^. Dental biofilm, collected from supragingival or subgingival surfaces, is highly enriched in site-specific bacteria, including those linked to periodontal disease and metabolic alterations, but its collection can be more invasive and technically demanding^14^.

These differences in sampling approach can significantly affect DNA yield, microbial richness, and the relative abundance of key taxa—especially when downstream quantification relies on sensitive techniques like qPCR. Inconsistencies in collection timing (e.g., fasting state, time of day), participant instructions (e.g., pre-sampling rinsing or brushing), and storage conditions further compound this variability^11^.

Despite these known influences, many studies do not compare sampling methods or account for their impact on microbiome profiles^15^. As a result, inter-study comparisons become difficult, and true biological signals may be covered or inflated due to methodological bias^15^. Standardizing and evaluating oral sampling techniques is therefore essential to ensure accurate, reproducible, and clinically meaningful microbiome data^16^. Moreover, few studies have considered how methodological variability may influence the biological relevance of the data—specifically, the ability to detect meaningful differences between clinical or phenotypic groups^17^. This represents a notable gap in the literature.

For instance, in oral microbiome studies comparing eutrophic (normal weight) and overweight/obese individuals, notable differences are often observed in the relative abundance of the phyla *Bacillota* and *Bacteroidota*^18^. Overweight and obese individuals typically exhibit an increased abundance of *Bacillota*—including genera such as *Streptococcus*, *Veillonella*, and *Lactobacillus*—which are associated with carbohydrate metabolism and acid production^18,19^. In contrast, *Bacteroidota*, which include genera like *Prevotella* and *Porphyromonas*, are generally found in lower abundance in overweight/obese individuals, though some variation exists depending on oral hygiene and inflammatory status. These shifts often result in a higher *Bacillota*-to-*Bacteroidota* ratio in obese individuals, a pattern that mirrors findings in gut microbiome research^19^. However, variability in sample site, age, and other confounding factors makes this trend less consistent in the oral cavity.

The present study aims to address this gap by evaluating the impact of different oral sample collection methods. This methodological study aimed to compare oral sample collection methods to identify the most consistent and reproducible approach for oral microbiome quantification by qPCR. In addition, to validate the biological applicability of the optimized method, the abundance of total bacteria (16 S rRNA gene), *Bacillota*, and *Bacteroidota* was assessed in samples obtained from eutrophic and overweight/obese individuals using the most consistent sampling method.

## Results

### Total bacterial load (16 S rRNA gene) and absolute abundance of phyla *Bacillota* and *Bacteroidota* per method

To assess the ideal collection method/sample (unstimulated saliva, cheek swab or biofilm) to perform oral microbiome analysis by qPCR, a total of 32 adolescents (16 females and 16 males; mean age 14 ± 2.4 years) were enrolled in this study.

**Fig. 1:**
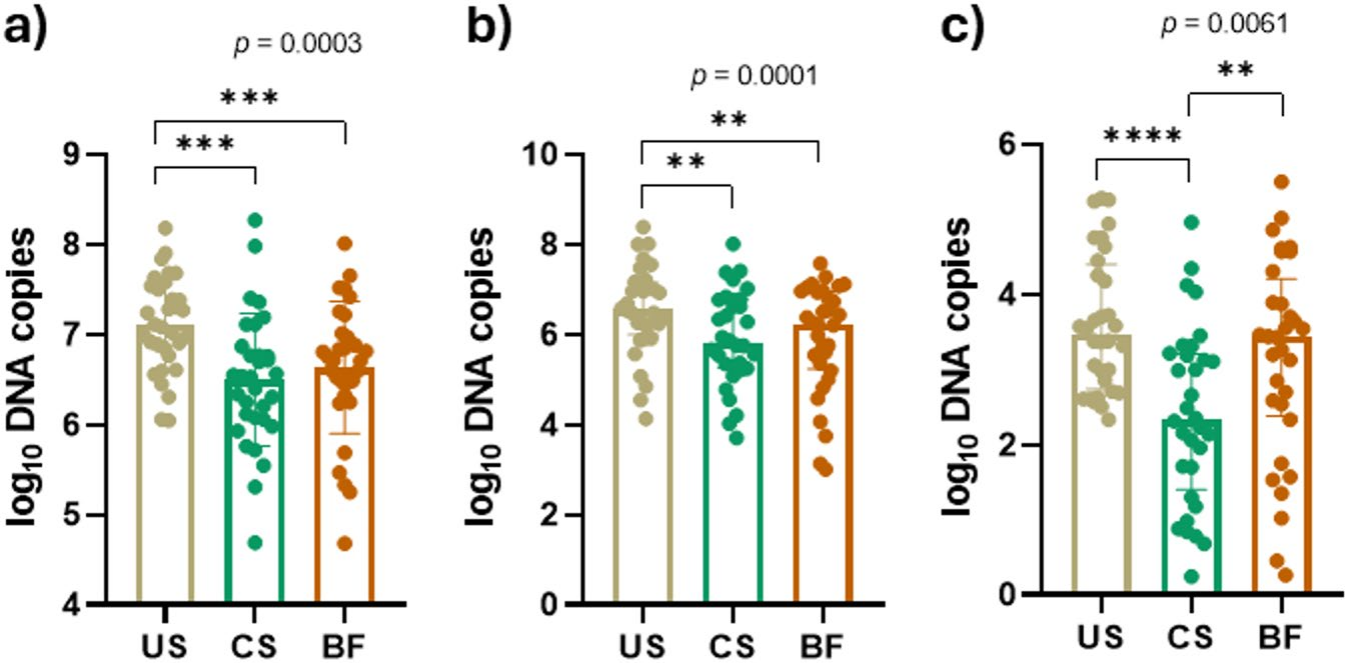
Comparison of different sample collection methods for oral microbiome analysis by qPCR (*n* = 32). Quantification of (**a**) 16 S rRNA gene; (**b**) *Bacillota*; (**c**) *Bacteroidota* is presented as Log₁₀-transformed DNA copy numbers. Bars indicate mean with SD for 16 S and median with interquartile range (IQR) for *Bacillota* and *Bacteroidota* phyla. Statistical analysis with p value derived from the repeated measures one-way ANOVA test followed by Tukey test for 16 S and Friedman test followed by Dunn test for both bacterial phyla. To assess the statistical significance between two independent groups the paired t test (16 S) and Wilcoxon test (*Bacillota*; *Bacteroidota*) were used. **p ≤ 0.01; ***p ≤ 0.001; ****p ≤ 0.0001. US: unstimulated saliva; CS: Cheek Swab; BF: Biofilm.

DNA samples extracted from the three samples collected for each individual were used for the qPCR analysis of the total bacterial load (16 S rRNA gene) and the phyla *Bacillota* and *Bacteroidota.* The results showed that unstimulated saliva yielded the most consistent and least variable measurements compared with cheek swab and biofilm samples (Fig. 1). Significant differences were observed among the collection methods for each bacterial group, with pairwise comparisons confirming that unstimulated saliva differs significantly from both cheek swab and biofilm samples. Quantification of *Bacteroidota* (Fig. 1c) exhibited the highest variability across collection methods; however, unstimulated saliva proved to be the most reliable and consistent approach, providing the highest yield, the lowest variability, and the most stable absolute abundance.

### Correlation of bacterial DNA copy numbers across collection methods for total bacteria, *Bacillota*, and *Bacteroidota*

**Fig. 2:**
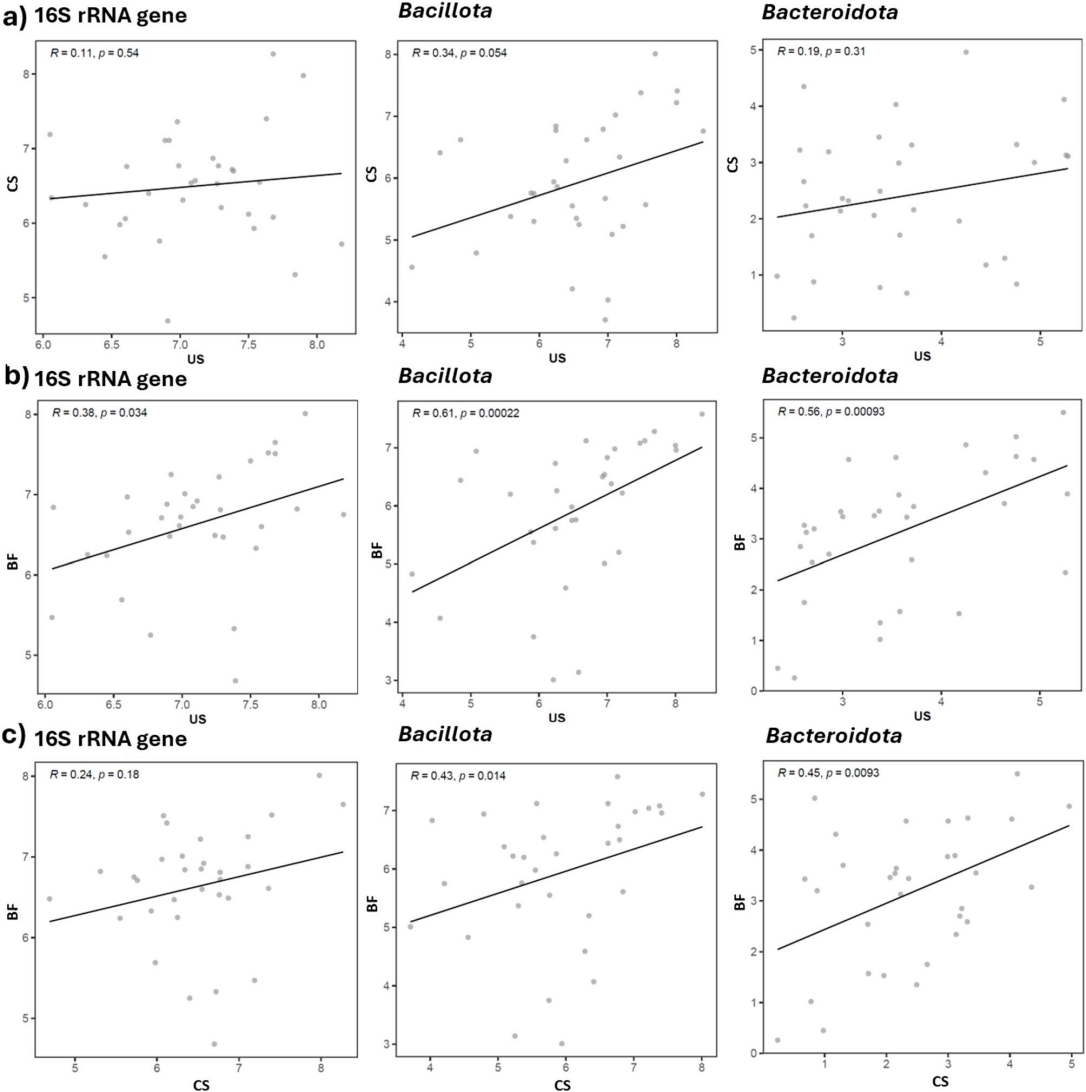
Scatterplots comparing log_10_-transformed bacterial 16 S rRNA gene, *Bacillota* and *Bacteroidota* copy numbers measured by different collection methods from the same individuals. Each gray dot represents an individual sample. The black line represents the linear regression fit. The association between two collection methods across distinct target regions (16 S rRNA gene, *Bacillota* and *Bacteroidota*) was assessed by Pearson or Spearman correlation coefficient (R) and its respective p-value. US: unstimulated saliva; CS: Cheek Swab; BF: Biofilm.

To evaluate the correlation of DNA copy numbers obtained by the different collection methods, correlation coefficients (R) and corresponding *p*-values were calculated for each target genomic region within the same individuals (Fig. 2). DNA copy numbers from unstimulated saliva and cheek swab (Fig. 2A) samples showed weak or negligible correlations across all analyzed regions (16 S rRNA gene, *Bacillota*, and *Bacteroidota*), indicating poor consistency between these methods. In contrast, unstimulated saliva and biofilm samples (Fig. 2B) exhibited moderate and statistically significant correlations, particularly for *Bacillota* and *Bacteroidota*, suggesting modest agreement in bacterial quantification by qPCR. Comparisons between cheek swab and biofilm samples (Fig. 2C) revealed weak correlations for total bacterial load and weak to moderate, yet significant, correlations for *Bacillota* and *Bacteroidota*. Overall, the unstimulated saliva and biofilm methods demonstrated the highest consistency in bacterial DNA quantification by qPCR.

### Comparative profile of 16 S rRNA gene, *Bacillota* and *Bacteriodota* between eutrophic and overweight/obese individuals using unstimulated saliva samples

Given that unstimulated saliva proved to be the most consistent collection method—yielding the lowest variability and most stable absolute abundance—saliva samples were used to compare oral microbiota composition (16 S rRNA gene, *Bacillota*, and *Bacteroidota*) between eutrophic and overweight/obese individuals. The demographic characteristics and body composition parameters (BMI, fat mass, and lean mass) of the study population are summarized in Table 1. Participants were classified into two groups: eutrophic and overweight/obese. No significant differences in age or gender distribution were observed between the groups, indicating a comparable demographic composition. As anticipated, body composition parameters differed significantly according to nutritional status, with higher BMI and fat mass and lower lean mass observed among overweight/obese individuals (*p* < 0.0001).

**Table 1:** Study population characteristics according to nutritional status.

	Eutrophic (*n* = 18)	Overweight/Obese (*n* = 18)	*p*-value
Age (years)	13.72 ± 2.47	13.50 ± 2.64	0.7958^a^
Gender (F/M)	8/10	8/10	> 0.9999^b^
BMI	18.33 ± 2.02	25.27 ± 4.83	< 0.0001^a^
FM (%)	24.01 ± 6.35	35.20 ± 6.67	< 0.0001^a^
LM (%)	75.99 ± 6.35	64.34 ± 6.61	< 0.0001^a^

Age, BMI (Body Mass Index), FM (Fat Mass) (%) and LM (Lean Mass) (%) values are reported as mean (SD).

^a^Independent-Samples T Test.

^b^Fisher’s exact test.

**Fig. 3:**
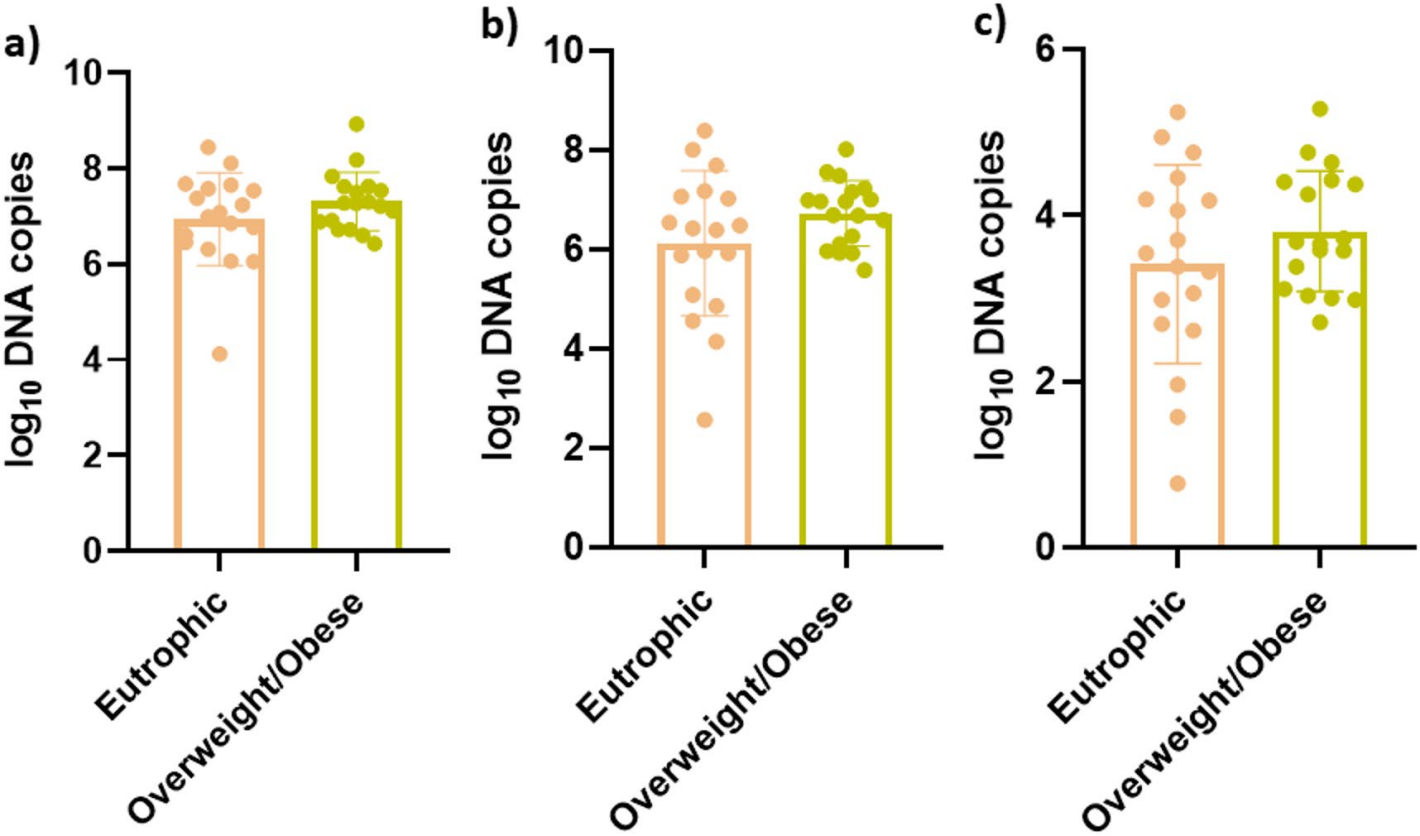
Comparison of bacterial DNA copy numbers in unstimulated saliva samples from eutrophic and overweight/obese individuals. Log₁₀-transformed DNA copy numbers for (**a**) 16 S rRNA gene), (**b**) *Bacillota*, and (**c**) *Bacteroidota* were quantified by qPCR in saliva samples collected from eutrophic (*n* = 18) and overweight/obese (*n* = 18) participants. Bars indicate mean with SD. Statistical comparisons were performed using an unpaired t-test with Welch’s correction; no significant differences were observed (*p* > 0.05).

Despite the clear differences in body composition parameters between the two groups, the overweight/obese participants exhibited a trend toward higher DNA copy numbers across the analyzed bacterial taxa compared with the eutrophic group (Fig. 3).

**Fig. 4:**
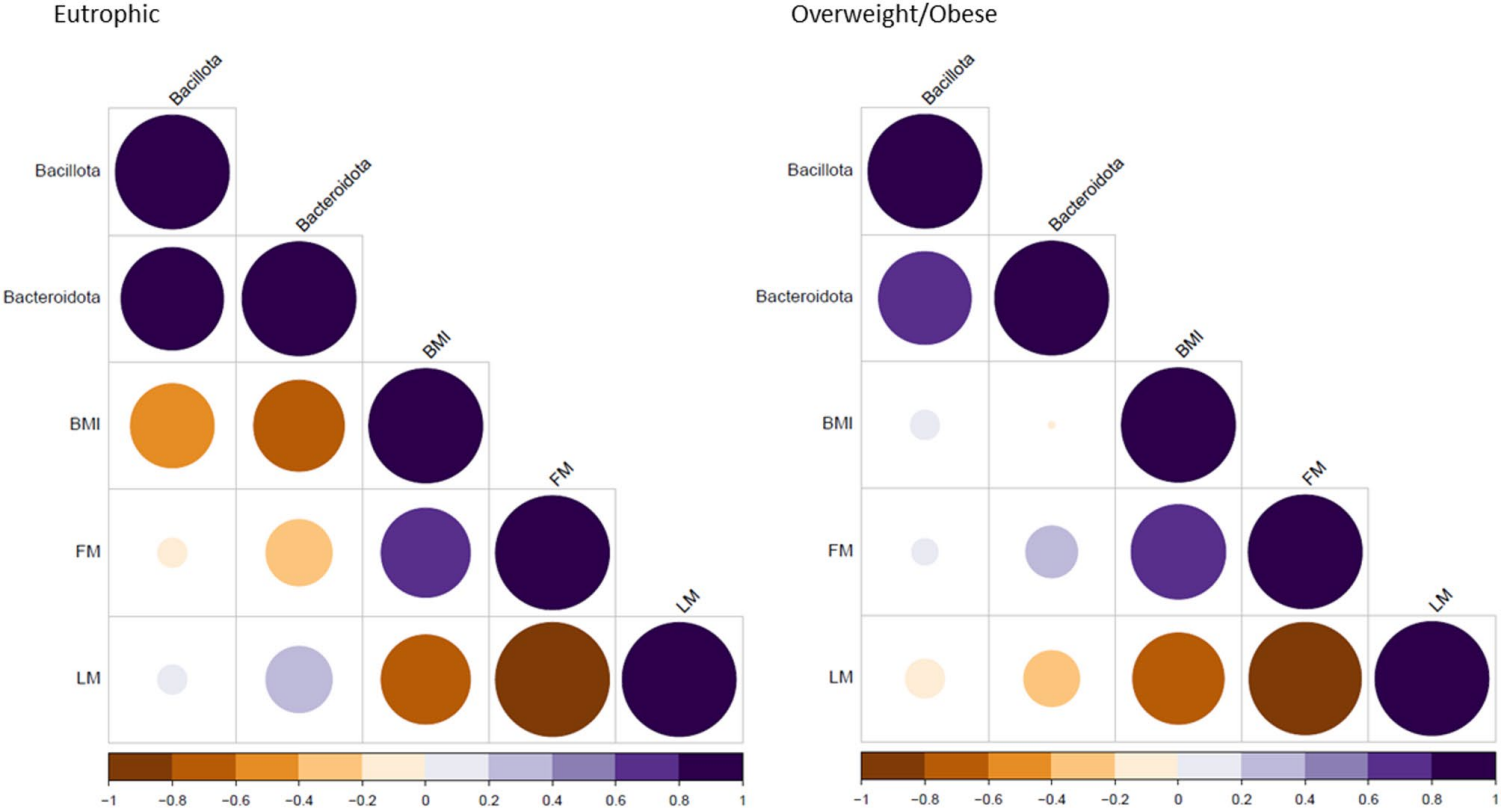
Correlation matrix of the study variables (*Bacillota*, *Bacteroidota*, BMI, FM and LM) in Eutrophic (left panel) and overweight/obese (right panel) individuals using a color-coded and size-scaled correlation plot. Pearson correlation coefficients are represented by color (blue= positive, brown = negative; white = near zero), and the size of each circle reflects the statistical significance of the correlation (larger circles indicate lower p-values). BMI: Body Mass Index; FM: Fat Mass (percentage); LM: Lean Mass (percentage).

To further assess the correlation between the different bacterial taxa and the parameters like body mass index (BMI), fat mass (FM) and lean mass (LM) in eutrophic and overweight/obese individuals, a correlation matrix was generated and is presented in Fig. 4; Tables 2 and 3. Correlation analysis of oral microbiota and body composition parameters revealed distinct patterns between eutrophic and overweight/obese individuals. In general, the eutrophic and overweight/obese groups exhibited inverse associations of *Bacillota* and *Bacteroidota* abundances with body composition parameters. For example, *Bacillota* showed a positive correlation with adiposity measures (BMI and FM) in overweight/obese individuals and a negative correlation with these metrics in eutrophic individuals. An identical pattern for *Bacteroidota* in eutrophic individuals with stronger and statistically significant correlations compared to the *Bacillota* phylum was observed. Similar but inverse results (except for BMI) for the *Bacteroidota* phylum in the overweight/obese group were also observed. These findings suggest that oral microbiota composition may reflect or contribute to differences in body composition parameters.

**Table 2:** Pearson correlation coefficients (r) and p-values between* Bacillota* and* Bacteroidota* and body composition parameters [BMI, fat mass (FM), and lean mass (LM)] in the Eutrophic group.

	*Bacillota*		*Bacteroidota*		BMI		FM		LM	
	*r*	*p*-value	*r*	*p*-value	*r*	*p*-value	*r*	*p*-value	*r*	*p*-value
*Bacillota*	1.00	NA	0.81	≤ 0.0001	− 0.54	0.0206	− 0.07	0.7941	0.07	0.7941
*Bacteroidota*	0.81	≤ 0.0001	1.00	NA	− 0.63	0.0048	− 0.34	0.1702	0.34	0.1702
BMI	− 0.54	0.0206	− 0.63	0.0048	1.00	NA	0.61	0.0068	− 0.61	0.0068
FM	− 0.07	0.7941	− 0.34	0.1702	0.61	0.0068	1.00	NA	− 1.00	≤ 0.0001
LM	0.07	0.7941	0.34	0.1702	− 0.61	0.0068	− 1.00	≤ 0.0001	1.00	NA

**Table 3:** Pearson correlation coefficients (r) and p-values between *Bacillota* and *Bacteroidota* and body composition parameters [BMI, fat mass (FM), and lean mass (LM)] in the overweight/obese group.

	*Bacillota*		*Bacteroidota*		BMI		FM		LM	
	*r*	*p*-value	*r*	*p*-value	*r*	*p*-value	*r*	*p*-value	*r*	*p*-value
*Bacillota*	1.00	NA	0.66	0.0027	0.07	0.7971	0.05	0.8305	− 0.12	0.6420
*Bacteroidota*	0.66	0.0027	1.00	NA	0.00	0.9885	0.21	0.4102	− 0.24	0.3358
BMI	0.07	0.7971	0.00	0.9885	1.00	NA	0.69	0.0016	− 0.64	0.0040
FM	0.05	0.8305	0.21	0.4102	0.69	0.0016	1.00	NA	− 0.97	≤ 0.0001
LM	− 0.12	0.6420	− 0.24	0.3358	− 0.64	0.0040	− 0.97	≤ 0.0001	1.00	NA

## Discussion

This study was designed as a methodological evaluation of different oral sampling techniques for microbiome analysis by qPCR, with the aim of identifying a reliable and standardized approach for bacterial quantification. By comparing saliva, cheek swab, and biofilm samples collected from the same individuals, this study demonstrated that unstimulated saliva provides the most consistent and reproducible results with lower variability in the quantification of the 16 S rRNA gene, *Bacillota*, and *Bacteroidota*^20^.

The primary goal was not to investigate disease-related differences but to validate a sampling method capable of supporting reproducible oral microbiome research. The secondary objective was an exploratory comparison between eutrophic and overweight/obese participants to assess the biological applicability of the saliva-based approach. Although no statistically significant differences were observed between groups, the results illustrate the method’s robustness and its potential to capture biologically relevant variation in larger or longitudinal studies. Methodological consistency represents a critical attribute for reliable microbiome assessment and biologically expected differences between oral niches do not compromise the methodological comparisons performed.

The comparison of total bacterial load and phylum-level abundance revealed significant differences among collection methods. Unstimulated saliva consistently produced higher and more reproducible quantifications of the 16 S rRNA gene, *Bacillota*, and *Bacteroidota*, while *Bacteroidota* exhibited the greatest variability across methods. These findings indicate that saliva not only maximizes bacterial recovery but also minimizes methodological noise, which is essential for reproducible microbiota analyses. The pairwise comparisons further confirmed that saliva-derived measurements differed significantly from those obtained by cheek swab or biofilm sampling, emphasizing that, as suggested by other authors, the choice of collection method can markedly influence the resulting microbial profile^15,21^.

The moderate correlation observed between unstimulated saliva and biofilm samples suggests that both methods capture overlapping microbial communities, though saliva offers greater consistency and ease of collection. Conversely, the weak correlation between saliva and swab samples indicates that sampling site/method strongly influences bacterial recovery and quantification^22^. These results align with previous reports highlighting that the oral microbiota is heterogeneous across different niches^23,24^, reinforcing the value of saliva as a composite and representative sample of the oral ecosystem. For studies dealing with localized processes, such as caries, periodontal disease or mucosal alterations, sample collection methods which reflect the “local” communities may be more informative^25^.

Previous studies have shown that there is an important association between oral bacterial composition and overweight and obesity^23,26–28^. For example, the systematic review from Lemos and colleagues^18^ reinforced this association between oral bacteria composition and nutritional status in children and adolescents.

The results presented here show no statistically significant differences in the abundance of *Bacillota* and *Bacteroidota* between eutrophic and overweight/obese individuals, although the overweight/obese group showed a tendency toward higher copy numbers.

The absence of statistically significant differences between eutrophic and overweight/obese adolescents should be interpreted with caution. Given the exploratory nature of this comparison, the limited sample size, and the low taxonomic resolution (phylum-level analysis), the study may have been underpowered to detect subtle biological differences. Importantly, non-significant findings do not necessarily indicate the absence of microbiome-related effects but may reflect methodological and biological variability inherent to complex microbial ecosystems.

Nonetheless, the consistent performance of the unstimulated saliva method reinforces its applicability for the characterization of microbial taxa across diverse research contexts, including studies exploring how the oral microbiome reflects physiological traits such as metabolism^29^, nutritional status^18^ and overall health^30^. Correlation analyses further indicated potential associations between bacterial taxa and body composition parameters^31–34^, aligning with previous evidence linking *Bacillota* and *Bacteroidota* to metabolic regulation and energy balance^27,31,35^. For instance, these results herein presented agree with the study from Stefura and colleagues^32^, who suggest that patients with higher BMI must exhibit a different microbiota composition compared to patients fitting the normal BMI criteria. These observations suggest that microbiota can have an important role in the development of obesity. Although anthropometric measures such as BMI remain the standard approach for obesity classification, microbial quantification provides complementary biological information. Bacterial load measurement does not aim to replace traditional clinical metrics but rather to characterize microbial ecology and its potential relationships with host physiology. In this context, microbial quantification may contribute to understanding host–microbiome interactions, dysbiosis, and metabolic regulation, which cannot be inferred from anthropometric parameters alone.

Despite the methodological strengths of this study, certain limitations should be considered when interpreting the findings. First, the sample size was relatively modest. However, the primary objective of this work was methodological rather than population-based inference. The use of paired samples from the same individuals allowed minimization of inter-individual variability, thereby providing a robust framework for comparing sampling approaches. Nevertheless, larger cohorts would enhance statistical power for detecting subtle biological differences.

DNA extraction was performed using the Salting Out method. Although commercial extraction kits are frequently employed in microbiome studies and may provide optimized lysis efficiency, different extraction protocols can influence DNA yield and microbial representation. No direct comparison with commercial kits was performed, as the study prioritized methodological consistency across sampling approaches. Importantly, DNA suitability was functionally supported by consistent qPCR amplification efficiency. Future studies may benefit from systematic comparisons of extraction methodologies.

Finally, given that oral microbiota composition undergoes age-dependent changes, the findings of this study are specific to an adolescent population and should not be directly extrapolated to adult cohorts. Future studies may explore whether similar methodological patterns are observed across different age groups.

Despite the limitations of the study our results show that this qPCR-based strategy offers a simple and low-cost approach that can yield robust and reliable microbiological data, which is particularly relevant for large-scale or population-based studies where practicality and standardization are essential, facilitating simpler data analysis and supporting its potential use for future point-of-care applications in oral and systemic health monitoring.

Future studies should expand this approach by including larger and more heterogeneous cohorts and expanding the range of bacterial targets quantified by qPCR to improve taxonomic resolution and sensitivity. Such work will further assess saliva as a versatile and standardized biological matrix for oral microbiome assessment, enabling its use in nutritional, metabolic, and systemic health research.

Among the oral sampling methods evaluated, unstimulated saliva demonstrated the highest reproducibility in bacterial quantification, confirming its suitability as a standardized biological matrix for oral microbiome studies.

Although exploratory comparisons between eutrophic and overweight/obese individuals showed no significant differences between nutritional groups, modest correlations with body composition parameters suggest potential links between oral bacteria and host physiology. Nevertheless, these analyses validate the biological applicability of saliva-based methods, providing a bridge between fundamental microbiome science and applied health research.

## Materials and methods

### Study design

The present study design was a methodological study, including only adolescent participants seen at the Adolescent Outpatient Clinic - Centro Integrado de Saúde Amaury de Medeiros (CISAM), located in the city of Recife-Pernambuco-Brazil. Participants were recruited if they met the eligibility criteria described below. The participants who agreed to participate in this study were notified about the protocol and procedures and signed a written consent form and filled out a survey. The study included a single visit, and each participant contributed a single sample. The Ethics Committee (Ethics Committee Approval Number: 4.865.118 and CAAE: 42220719.6.0000.5207) approved this study protocol. The study was performed in conformity with the Helsinki Declaration of 1975 (revised in 2013).

The inclusion criteria for the study were adolescents of both sexes aged between 10 and 19 years, 11 months, and 29 days. The exclusion criteria were adolescents with physical and/or mental disabilities, pregnant girls, oral cavity problems that prevented the collection of biological material (gingivitis, cavities, bleeding gums).

### Collection of biological material

Biological samples from the oral cavity were collected as previously described by our group between eight and ten o’clock in the morning due to circadian events in salivary flow^36,37^. The adolescents were asked not to eat, drink, or perform oral hygiene one hour before collection. The collection was carried out using three different methods: drooling to collect unstimulated saliva (US), oral cytological brushing (swab) to collect cells from the cheek mucosa (CS), and toothpick to collect oral biofilm (BF).

The first sample collected was unstimulated saliva: participants were instructed to refrain from swallowing for two minutes, after which, with assistant from a research team member, approximately 1 mL of saliva was drooled into a sterile 1.5 mL plastic microtube, which was later placed on ice^38^.

In the cheek mucosa cells cytology brush collection, the procedure involved brushing the inner surface of each cheek with a sterilized cytology brush, performing 30 circular motions per side. After sampling, the outer portion of the brush stem was cut off and placed into a 2.0 mL plastic microtube containing 1.0 mL of absolute ethanol^39^. Finally, the oral biofilm was collected from the interdental area with an autoclaved toothpick. This process was repeated and up to five toothpicks were collected and pooled in each tube. After the biofilm was removed, the toothpick was immersed in 2.0 mL plastic microtubes containing 1.0 mL of absolute ethanol^40^.

### Sample processing and storage

All samples were processed and stored at the Molecular Biology Laboratory of the Pediatric Oncohematology Center at Osvaldo Cruz University Hospital, University of Pernambuco. Unstimulated saliva was stored at −80 °C and biofilm and cheek swab samples were kept at −20 °C until DNA extraction.

### DNA extraction

DNA extraction was performed using an operational protocol based on the Salting Out method, which promotes protein removal through high salt concentrations, allowing the isolation of high-purity DNA without the use of toxic organic solvents^41^. After extraction, DNA samples were quantified in the nanodrop to determine their concentrations. DNA samples, previously diluted in 20 µL of water, were subjected to lyophilization using a Terroni benchtop LS 3000 freeze dryer (Terroni Instruments, Sao Paulo, Brazil) according to the manufacturer instructions. This equipment offers a useful shelf area ranging from 0.5 to 45 m², allowing adaptation to different production needs. During the process, the samples were frozen and subsequently exposed to controlled vacuum sublimation, ensuring complete water removal and preservation of the integrity of the lyophilized DNA. DNA integrity following lyophilization was functionally supported by consistent qPCR amplification efficiency (Figure S1).

### RT-qPCR

The absolute abundance of 16 S rRNA gene and the bacterial phyla *Bacillota* and *Bacteroidota* per collection method was determined by quantitative real-time PCR (qRT-PCR). To accomplish this, universal primers targeting bacterial 16 S rRNA gene (926 F: 5′ AAACTCAAAKGAATTGACGG 3′; 1062R: 5′ CTCACRRCACGAGCTGAC 3′) and specific primers for *Bacillota* (928 F-firm: 5′ TGAAACTYAAGGAATTGACG 3′; 1040FirmR: 5′ACCATGCACCACCTGTC 3′) and *Bacteroidota* (798cfbF: 5′ CRAACAGGATTAGATACCCT 3′; cfb967R: 5′ GGTAAGGTTCCTCGCGCTAT 3′) were used. Primer sequences were obtained from Bacchetti De Gregoris et al., 2011^42^. Primers were selected based on their prior validation in quantitative PCR studies, reported amplification efficiency, and specificity for the targeted bacterial groups. The chosen primer sets target conserved regions of the 16 S rRNA gene, enabling reliable quantification at the phylum level. Although bacterial taxonomy and phylogenetic classifications continue to evolve, the primers employed in this study remain widely used and appropriate for comparative quantitative analyses. Importantly, the study’s methodological focus prioritizes measurement consistency rather than fine-scale taxonomic resolution.

Each DNA sample was analysed in duplicate in a PCR reaction of a total volume of 10 µL using the NZYSpeedy qPCR Green Master Mix (2x) (MB224, NZYtech, Lisboa, Portugal) with 0.4 µM of each forward and reverse primers. To normalize variations in DNA yield between samples, which may occur during extraction and to ensure a consistent starting point for amplification a standardized amount of 1ng DNA per PCR reaction was used. No-template controls (NTCs) were included in all qPCR runs to assess potential contamination and non-specific amplification.

Amplification and detection of DNA by qPCR were conducted with the CFX96 Touch Real-Time PCR Detection System (Bio-Rad, Berkeley, CA, USA) according to the following PCR conditions: 95 °C for 3 min and 40 cycles of 95 °C for 5 s and 61.5 °C for 20 s. To perform absolute quantification of total bacterial load (16 S rRNA gene) and *Bacillota* and *Bacteroidota* by qRT-PCR, standards for these bacterial assays were prepared according to the protocol described before^43^. Duplicate tenfold dilutions of plasmid DNA corresponding to seven nonzero standard concentrations were used and ranged from 2.77 × 10^10^ to 2.77 × 10^4^ copies of DNA per reaction for 16 S rRNA, 1.44 × 10^8^ to 1.44 × 10^2^ for *Bacteroidota*, and 1.74 × 10^9^ to 1.74 × 10^3^ for *Bacillota*. The DNA copy numbers of the 16 S rRNA gene and *Bacillota* and *Bacteroidota* phyla within each sample were calculated from the standard curve. To facilitate the accuracy and interpretability of results, DNA copy numbers were Log_10_-transformed. Consistency was evaluated based on the dispersion (variability) of Log10-transformed bacterial DNA copy numbers obtained with each sampling method.

### Anthropometric and body composition indicators

Height and weight were measured according to the procedures detailed by Jellife (1968) and the World Health Organization (1995). Weight was measured using a Líder^®^ scale, model P-200 C, ID: LD1050, with a maximum capacity of 200 kg and accuracy of 100 g. Participants were weighed barefoot and wearing light clothing. Height was measured using a stadiometer fixed to a flat surface (a wall without baseboards). Participants stood upright, maintaining contact with the wall at five anatomical points (heels, calves, buttocks, shoulders, and head) while looking straight ahead toward the horizon. The measuring equipment was calibrated daily during data collection. Body Mass Index (BMI) was calculated using the formula BMI = weight (kg)/height (m²) to assess nutritional status. The classification was determined based on z-scores calculated with the WHO AnthroPlus software (version 1.0.4). Nutritional status categories were defined as follows: Severe Thinness, Z-score < −3; Thinness, Z-score ≥ −3 and < −2; Eutrophic, Z-score ≥ −2 and ≤ + 1; Overweight, Z-score > + 1 and ≤ + 2; and Obesity, Z-score > + 2^44^. In this study, adolescents classified as overweight or obese were combined into a single category designated as overweight/obese.

The body composition was assessed using bioelectrical impedance analysis (BIA 1010, Sanny, São Paulo, Brazil). This procedure provides data on total body water (in liters and percentages), fat-free mass (in kg and percentage), body fat (in kg and percentage), basal energy expenditure, and total energy expenditure. Equations specific to children and adolescents proposed by Kushner et al., 1992^45^and Houtkooper et al., 1992^46^ were applied. Body fat percentage was evaluated according to the criteria of McCarthy et al., 2006^47^, which considers age and the corresponding percentile. The 2nd, 85th, and 95th percentiles define the cutoff points for undernutrition, overweight, and obesity.

### Statistical analysis

No formal power calculation was performed. The study was primarily designed as a methodological evaluation of oral sampling approaches, where paired measurements within the same individuals were used to assess variability and reproducibility. The comparison between eutrophic and overweight/obese individuals was exploratory and intended to assess the biological applicability of the optimized sampling method rather than to detect predefined differences between groups.

For normally distributed data, descriptive statistics are presented as mean ± standard deviation, for non-normally distributed data are given as median with interquartile range (IQR) and for the qualitative variables data is presented as frequencies. The choice between parametric and non-parametric tests was based on data distribution. Normality was evaluated using the Shapiro–Wilk test. Variables meeting normality assumptions were analyzed using parametric tests, while variables violating these assumptions were analyzed using non-parametric methods.

For categorical data, Fisher’s exact test was utilized to determine the relationship between the qualitative variables among the groups under study. Regarding the statistical analysis of continuous variables, an Independent-Samples T Test was used. In further statistical analysis, a paired t test for normally distributed data and the Wilcoxon signed-rank test for non-normally distributed data were used. For unpaired data the unpaired *t*-test with Welch’s correction was used. For differences comparison among groups the repeated measures one-way ANOVA test (parametric) followed by Tukey test and the Friedman test (non-parametric) followed by Dunn test were conducted. To assess the pairwise correlation coefficients (R) and corresponding *p*-values of the log_10_-transformed bacterial copy numbers across different collection methods, the Pearson or Spearman correlation tests were used. In a similar way, the correlation matrix of the study variables in eutrophic and overweight/obese individuals using the Pearson correlation test and corresponding p-values was also generated. The chance of incorrectly rejecting a true null hypothesis was set up to *p* ≤ 0.05.

Statistical analyses and graphic presentations were executed using the commercially available GraphPad Prism 8 software and R software version 4.4.1.

## Supplementary Information

Below is the link to the electronic supplementary material.

Supplementary Material 1

## Data Availability

The data used to generate and support the findings of this study are available from the corresponding author upon request.
