# Supplementary material for: Standardizing oral microbiome sampling for qPCR: methodological and exploratory insights into nutritional status
Source: Sci Rep. 2026 Mar 14;16:13501. doi: 10.1038/s41598-026-43909-7 (PMC13111650; doi:10.1038/s41598-026-43909-7)
Supplement: Supplementary file 1 — Supplementary Material 1 [file 41598_2026_43909_MOESM1_ESM.docx]

**SUPPLEMENTARY FIGURE**


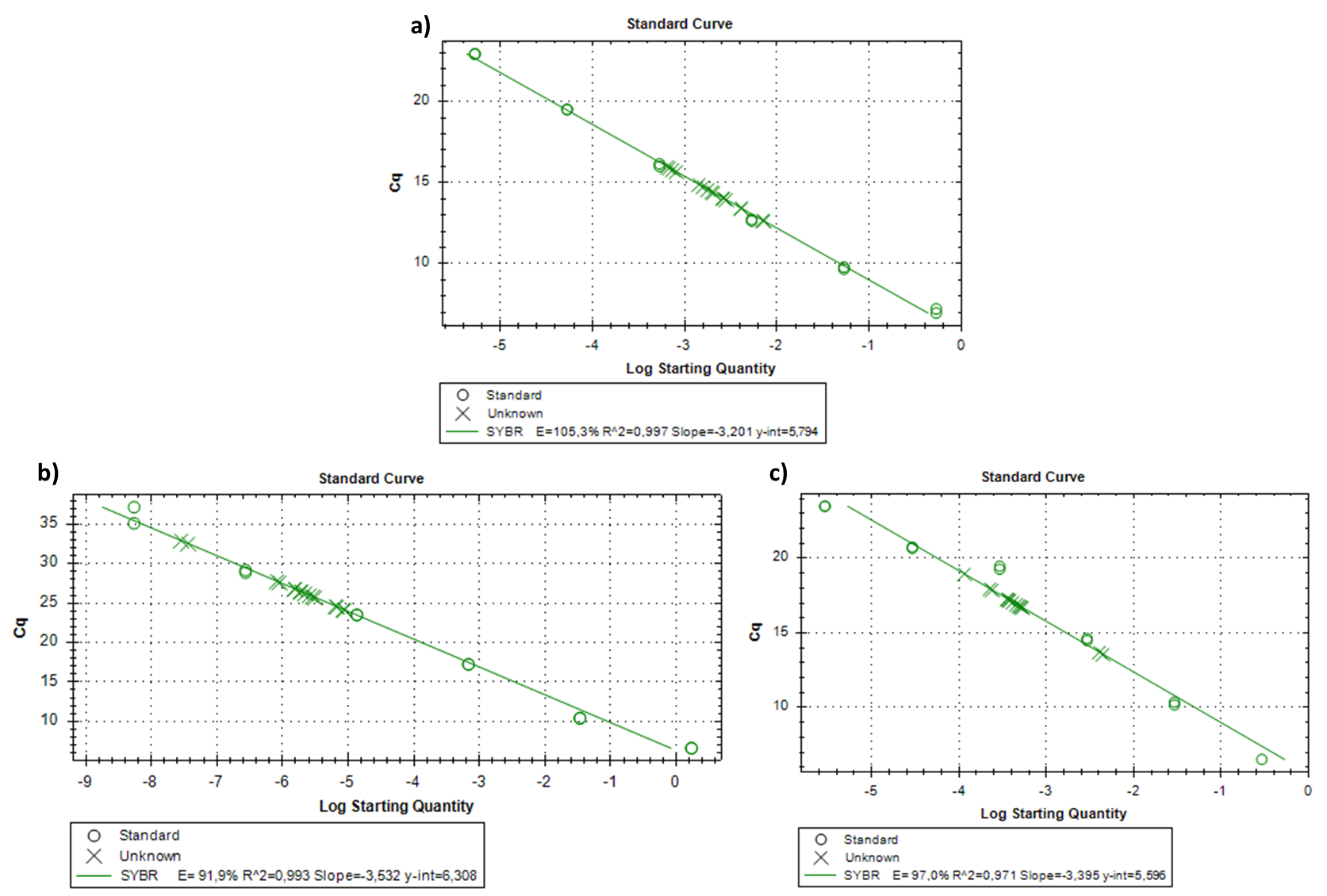


**Supplementary Figure S1.** Standard curves and RT-qPCR amplification efficiencies for 16S rRNA a), Bacteroidota b), and Bacillota c). The Ct values were plotted against the logarithm of template concentration to assess assay performance. Amplification efficiencies (E) and correlation coefficients (R²) were calculated from the slope of each curve. All three assays demonstrated high linearity and efficiency within the acceptable range (90–110%), indicating reliable quantification of each microbial group.
